# BEHAVIORAL INTERVENTIONS FOR COGNITION AND EVERYDAY FUNCTION: A SYSTEMATIC REVIEW

**DOI:** 10.1093/geroni/igz038.2420

**Published:** 2019-11-08

**Authors:** Briana N Sprague, Sara A Freed, Christina E Webb, Christine B Phillips, Jinshil Hyun, Lesley A Ross

**Affiliations:** 1 The Pennsylvania State University, University Park, Pennsylvania, United States; 2 University of Texas at Dallas, Dallas, Texas, United States; 3 Arizona State University, Phoenix, Arizona, United States; 4 Albert Einstein College of Medicine, New York, New York, United States

## Abstract

Behavioral interventions to improve cognitive function in older adults are widespread and can vary from theatre classes to cognitive training programs targeting one domain. However, the effectiveness in maintaining different cognitive domains varies greatly both across and within intervention types. To date, no systematic reviews have synthesized findings across more than a few types of interventions (e.g., cognitive vs. exercise). This systematic review examined nine types of behavioral interventions and the respective transfer to 18 cognitive domains and everyday function. The 2017 search yielded 75 unique eligible articles comprising of educational, theatre, mindfulness, cognitive, exercise, video game, and combination interventions. In general, there was limited evidence of consistent transfer from behavioral interventions to untrained cognitive domains. Few studies examined education, theatre, mindfulness, or video game interventions, leaving inconclusive results about their effect on cognitive function. Nine studies evaluated transfer to everyday function and found that both process- and strategy-based cognitive training conferred benefits up to 10 years posttest. These results suggest that while there is weak-to-moderate evidence of far transfer from behavioral interventions to untrained cognitive domains, it may be more important to examine far transfer to measures more indicative of older adult everyday life. Furthermore, it highlights the necessity to continue long-term follow-up. While there were notable limitations of the extant literature, including inconsistent use of terms such as active control or inadequate intervention description, there were strengths such as the recent implementation of factorial designs. Implications for future research and practice will be discussed.

